# COX-2 rs689466, rs5275, and rs20417 polymorphisms and risk of head and neck squamous cell carcinoma: a meta-analysis of adjusted and unadjusted data

**DOI:** 10.1186/s12885-016-2535-3

**Published:** 2016-07-13

**Authors:** Wei-Dong Leng, Xiu-Jie Wen, Joey S. W. Kwong, Wei Huang, Jian-Gang Chen, Xian-Tao Zeng

**Affiliations:** Department of Stomatology, Taihe Hospital, Institute of Oral and Maxillofacial Surgery, Hubei University of Medicine, Shiyan, 442000 China; Department of Stomatology, Daping Hospital & Research Institute of Surgery, Third Military Medical University, Chongqing, 400042 China; Chinese Cochrane Center, Chinese Evidence-Based Medicine Center, Western China Hospital, West China Hospital, Sichuan University, Chengdu, 610041 China; Department of Stomatology, Zhuhai People’s Hospital, Zhuhai Hospital Affiliated with Jinan University, Zhuhai, 519000 China; Department of Stomatology, Center for Evidence-Based and Translational Medicine, Zhongnan Hospital of Wuhan University, 169 Donghu Road, Wuhan, 430071 China

**Keywords:** COX-2 rs689466, COX-2 rs5275, COX-2 rs20417, Polymorphism, Head and neck squamous cell carcinoma, Meta-analysis

## Abstract

**Background:**

Numerous case–control studies have been performed to investigate the association between three cyclooxygenase-2 (COX-2) polymorphisms (rs20417 (−765G > C), rs689466 (−1195G > A), and rs5275 (8473 T > C)) and the risk of head and neck squamous cell carcinoma (HNSCC). However, the results were inconsistent. Therefore, we conducted this meta-analysis to investigate the association.

**Methods:**

We searched in PubMed, Embase, and Web of Science up to January 20, 2015 (last updated on May 12, 2016). Two independent reviewers extracted the data. Odds ratios (ORs) with their 95 % confidence intervals (CIs) were used to assess the association. All statistical analyses were performed using the Review Manager (RevMan) 5.2 software.

**Results:**

Finally 8 case–control studies were included in this meta-analysis. For unadjusted data, an association with increased risk was observed in three genetic models in COX-2 rs689466 polymorphism; however, COX-2 rs5275 and rs20417 polymorphisms were not related to HNSCC risk in this study. The pooled results from adjusted data all revealed non-significant association between these three polymorphisms and risk of HNSCC. We also found a similar result in the subgroup analyses, based on both unadjusted data and adjusted data.

**Conclusion:**

Current results suggest that COX-2 rs689466, rs5275, and rs20417 polymorphisms are not associated with HNSCC. Further large and well-designed studies are necessary to validate this association.

## Background

Head and neck squamous cell carcinoma (HNSCC) is 1 of the disease burdens worldwide affecting eating, breathing, and appearance. Besides environmental risk factors, such as tooth loss [1], alcohol consumption [2], periodontal diseases [3], smoking [4], tooth brushing [5], and human papillomavirus (HPV) [6], genetic factors [7, 8] also play an significant role in the onset and development of HNSCC. Many polymorphisms have been identified associated with risk of HNSCC by meta-analyses, such as the hOGG1 Ser326Cys polymorphism [9], XRCC1 Arg194Trp polymorphism [10], ERCC2 rs1799793 and rs13181 polymorphisms [11]; however, some polymorphisms including XPD Asp312Asn polymorphism [12], TP53 codon 72 polymorphism [7], and VEGF gene polymorphisms [13] are not associated with HNSCC risk. Particularly within the same gene, theXRCC1gene for example, XRCC1 Arg194Trp polymorphism was associated with increased risk while Arg399Gln and Arg280His polymorphisms were not [10].

The human cyclooxygenase-2 (COX-2), the key enzyme in the conversion of arachidonic acid to prostatglandins, is located at chromosome 1q25.2-q25.3 and rs20417 (−765G > C), rs689466 (−1195G > A), and rs5275 (8473 T > C) are the three commonly investigated polymorphisms in the COX-2 gene [14, 15]. Now the association between COX-2 gene polymorphisms and risk of many cancers, such as hepatocellular carcinoma [16], colorectal cancer [17], breast cancer [18], prostate cancer [19], gastric cancer [20] were investigated by meta-analyses. COX-2 has been confirmed very low or no expression in normal human oral tissues, otherwise it was elevated in oral precancerous lesions and over-expressed in oral squamous cell carcinoma (OSCC) [21]. The elevated expression of COX-2 was presented to be correlated with malignant transformation, advancing clinical stage, and disease progression [22].

There are also many published studies that explored the association between COX-2 rs689466, rs5275, and rs20417 polymorphisms and risk of HNSCC. Unfortunately, the results of published studies were inconsistent and using a meta-analytic method to pool these results for obtaining a more precise result [23] is necessary. In this meta-analysis, we extracted and combined crude data and adjusted data.

## Methods

We reported this meta-analysis according to the Preferred Reporting Items for Systematic Reviews and Meta-Analyses (PRISMA) statement [24] and ethical approval is not necessary.

### Eligibility criteria

Cohort studies or case–control studies evaluating the risk of HNSCC in relation to COX-2 rs689466, rs5275, and/or rs20417 polymorphisms were considered for eligibility if they also met the following criteria: (1) the cancer was HNSCC, oral squamous cell carcinoma (OSCC), or laryngeal squamous cell carcinoma (LSCC) confirmed using microscopic examination; (2) the frequency of genotype distribution, adjusted odds ratios (ORs) and their 95 % confidence intervals (CIs), or the data that can calculate them were reported; (3) full-text were obtainable; (4) if 2 or more studies covered the same population, we included the study that contained most comprehensive information; (5) the published language is English or Chinese.

### Search strategy

We searched PubMed, Embase, and Web of Science up to January 20, 2015 (last updated on May 12, 2016) using the following search terms: head and neck, oral, oral cavity, pharyngeal, oropharynx, laryngeal, laryngopharyngeal, mouth, tongue, carcinoma, cancer, tumour, neoplasm, cyclooxygenase-2, COX-2, PTGs2, polymorphism, mutation, variant, and variation. We also screened reference lists of recent reviews, eligible studies, and published meta-analyses on related topics for additional eligible studies.

### Data extraction

The following data were extracted from all eligible studies by 2 authors independently and disagreements (κ = 0.96) were resolved by discussion: last name of the first author; publication year; country and ethnicity; genotyping method; source of control, number and genotyping distribution of cases and controls; adjusted OR and its 95 % CI; adjusted variables; and Hardy–Weinberg Equilibrium (HWE) for controls [25]. The meta-analysis reviewers were blind to the study author and institution of the studies undergoing review.

### Statistical analysis

The heterogeneity was assessed first using the Cochrane *Q* and *I*^2^ statistic [26]. The heterogeneity was considered acceptable if both *p* > 0.1 and *I*^2^ < 40 % and used the fixed effect model, otherwise the random effect model was used. For crude data, we used OR and its 95 % confidence interval (CI) to quantify the strength of association using the allele comparison, homozygote comparison, heterozygote comparison, dominant model, and recessive model genetic models. For adjusted data, we directly combined the relevant ORs and their 95 % CIs according to reported genetic models. We performed subgroup analyses based on ethnicity, site of cancer, and HWE status for controls. The sensitivity analysis was performed by switching the effect model. Publication bias was assessed by funnel plots if the number of included studies was more than 9. All statistical analyses were performed using Review Manager (RevMan) software (version 5.2 for Windows; Copenhagen: The Nordic Cochrane Centre, The Cochrane Collaboration).

## Results

### Study identification and characteristics

**Fig. 1:**
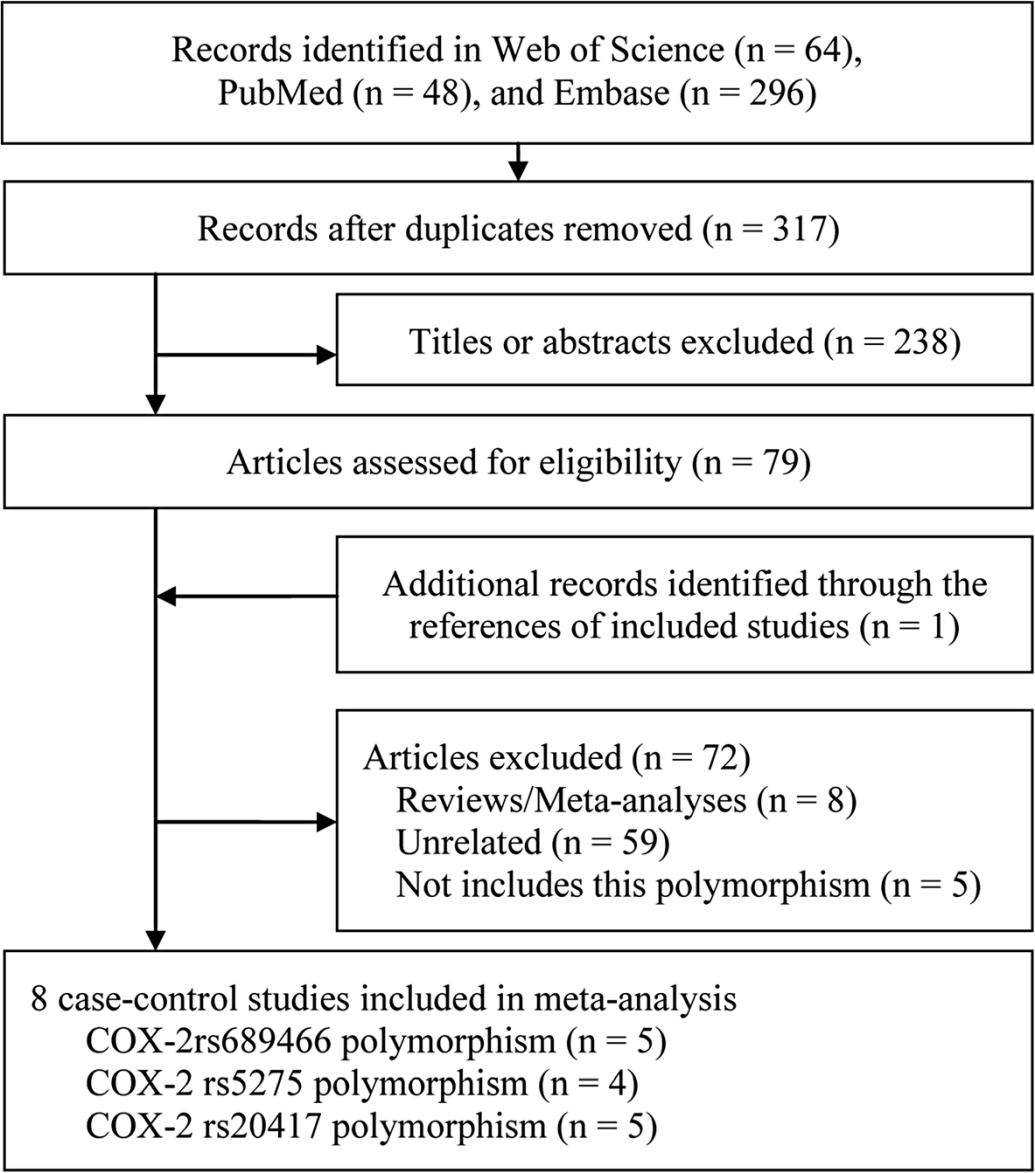
Study selection flowchart

We yielded 408 papers initially and 8 case–control studies [27–34] were included finally, Fig. 1 showed the progress of study selection. Of them, 5 case–control studies involving 1564 cases and 2346 controls focused on COX-2 rs689466 polymorphism [28, 30, 31, 33, 34], 4 studies involving 1259 cases and 2097 controls on COX-2 rs5275 polymorphism [27, 31, 33, 34], and 5 studies involving 1229 cases and 1164 controls on COX-2 rs20417 polymorphism [28–32]. One study did not satisfy the HWE for COX-2 rs5275 polymorphism [31] 1 for COX-2 rs20417 polymorphism [32]. The main characteristics are shown in Table 1 and Table 2.

**Table 1:** Characteristics and unadjusted data of included studies

Study	Country (Ethnicity)	Form of disease	Cases/Control				HWE	Smoking status	Genotyping methods
			Sample size	Genotype distribution					
rs689466 (−1195G > A)				GG	GA	AA			
Chiang 2008	China (Asian)	OSCC	368/441	80/114	187/235	101/92	Yes	Mixed	PCR-RFLP
Peters 2009	Netherlands (Caucasian)	HNSCC	431/438	275/260	134/163	22/15	Yes	Mixed	PCR
Mittal 2010	India (Asian)	OSCC	193/137	3/5	57/32	133/100	Yes	Smokers	PCR-RFLP
Chang 2013	China (Asian)	HNSCC	313/295	93/90	146/148	74/57	Yes	Mixed	Taqman
Niu 2014	China (Asian)	HNSCC	259/1035	61/222	126/542	72/271	Yes	Mixed	Taqman
		OSCC	140/1035	44/222	80/542	25/271	Yes	Mixed	Taqman
		LSCC	90/1035	17/222	46/542	27/271	Yes	Mixed	Taqman
rs5275 (8473 T > C)				TT	TC	CC			
Campa 2007	European (multicenter)	HNSCC	553/711	252/313	237/321	44/77	Yes	Mixed	TaqMan
		OSCC	252/711	113/313	117/321	22/77	Yes	Mixed	TaqMan
		LSCC	281/711	139/313	120/321	22/77	Yes	Mixed	TaqMan
Mittal 2010	India (Asian)	OSCC	135/59	74/24	53/34	8/1	No	Smokers	PCR-RFLP
Chang 2013	China (Asian)	HNSCC	313/295	209/199	89/86	15/10	Yes	Mixed	Taqman
Niu 2014	China (Asian)	HNSCC	258/1032	177/691	72/316	9/25	Yes	Mixed	Taqman
		OSCC	168/1032	118/691	45/316	5/25	Yes	Mixed	Taqman
		LSCC	90/1032	59/691	27/316	4/25	Yes	Mixed	Taqman
rs20417 (−765G > C)				GG	GC	CC			
Lin 2008	China (Asian)	OSCC	297/280	193/107	104/173	0/0	Yes	Mixed	PCR–RFLP
Chiang 2008	China (Asian)	OSCC	178/205	136/166	42/39	0/0	Yes	Mixed	PCR–RFLP
Peters 2009	Netherlands (Caucasian)	HNSCC	428/433	321/321	99/99	8/13	Yes	Mixed	PCR
Mittal 2010	India (Asian)	OSCC	176/96	92/41	78/49	6/6	Yes	Smokers	PCR–RFLP
Lakshmi 2012	India (Asian)	OSCC	150/150	110/142	28/6	12/2	No	Mixed	PCR–RFLP

*OSCC* oral squamous cell carcinoma; *HNSCC*, head and neck squamous cell carcinoma; *LSCC* laryngeal squamous cell carcinoma; *HWE* Hardy–Weinberg Equilibrium

**Table 2:** Adjustment and adjusted data of included studies

Study	Form of disease	Reference	OR (95 % CI)	Adjustment
rs689466 (−1195G > A)				
Peters 2009	HNSCC	GG: 1.00	GA: 0.79 (0.58–1.07);	age (continuous), sex, smoking (continuous, 5 levels), and alcohol consumption (continuous, 3 levels)
			AA: 1.24 (0.60–2.56)	
Mittal 2010	OSCC	GG: 1.00	GA: 3.07 (0.66–13.24);	age, gender
			AA: 2.22 (0.52–9.50)	
		G: 1.00	A: 1.03 (0.60–1.42)	
Chang 2013	HNSCC	GG: 1.00	GA: 0.86 (0.56–1.32);	sex, age, education, cigarette smoking (pack-year categories), betel quid chewing (pack-year categories), and alcohol drinking (frequency)
			AA: 1.23 (0.72–2.09);	
			GA + AA: 0.96 (0.64–1.43);	
		G: 1.00	A: 1.08 (0.83–1.40)	
Niu 2014	HNSCC	GG: 1.00	GA:0.85 (0.60–1.21)	age, sex, smoking status, and drinking status
			AA: 1.01 (0.69–1.50)	
			GA + AA: 0.91 (0.65–1.26)	
	OSCC	GG: 1.00	GA: 0.74 (0.49–1.11)	
			AA: 0.87 (0.55–1.39)	
			GA + AA:0.78 (0.53–1.14)	
	LSCC	GG: 1.00	GA:1.16 (0.65–2.09)	
			AA:1.43 (0.75–2.75)	
			GA + AA:1.23 (0.71–2.15)	
rs5275 (8473 T > C)				
Campa 2007	HNSCC	TT: 1.00	TC: 1.03 (0.82–1.28);	age, sex, center, tobacco consumption (packyears), and years of alcohol consumption
			CC: 0.75 (0.51–1.10);	
			TC + CC: 0.97 (0.78–1.20)	
	OPSCC	TT: 1.00	TC: 1.16 (0.86–1.58);	
			CC: 0.91 (0.53–1.54);	
			TC + CC: 1.11 (0.83–1.49)	
	LSCC	TT: 1.00	TC: 0.88 (0.63–1.22);	
			CC: 0.60 (0.34–1.05);	
			TC + CC: 0.82 (0.60–1.12)	
Mittal 2010	OSCC	TT: 1.00	TC: 0.27 (0.03–2.26);	age, gender
			CC: 0.28 (0.03–2.33)	
		T: 1.00	C: 0.88 (0.55–1.40)	
Chang 2013	HNSCC	TT: 1.00	TC: 1.04 (0.69–1.56);	sex, age, education, cigarette smoking (pack-year categories), betel quid chewing (pack-year categories), and alcohol drinking (frequency)
			CC: 1.89 (0.74–4.82);	
			TC + CC: 1.12 (0.75–1.65)	
Niu 2014	HNSCC	TT: 1.00	TC: 0.90 (0.66–1.22);	age, sex, smoking status, and drinking status
			CC: 1.48 (0.68–3.25);	
			TC + CC: 0.94 (0.70–1.26)	
	OSCC	TT: 1.00	TC: 0.86 (0.58–1.26);	
			CC: 1.03 (0.36–2.97);	
			TC + CC: 0.87 (0.60–1.27)	
	LSCC	TT: 1.00	TC: 1.02 (0.63–1.64);	
			CC: 1.62 (0.54–4.88);	
			TC + CC: 1.07 (0.67–1.69)	
rs20417 (−765G > C)				
Lin 2008	OSCC	GG: 1.00	GC + CC: 0.22 (0.12–0.39)	age, gender, ethnicity, educational level, and habits of betel quid chewing, cigarette smoking, and alcohol drinking
Peters 2009	HNSCC	GG: 1.00	GC: 0.99 (0.71–1.40);	age (continuous), sex, smoking (continuous, 5 levels), and alcohol consumption (continuous, 3 levels)
			CC: 0.59 (0.23–1.49)	
Mittal 2010	OSCC	GG: 1.00	GC: 0.71 (0.42–1.18);	age, gender
			CC: 0.44 (0.13–1.46)	
		G: 1.00	C: 0.73 (0.50–1.08)	

*OSCC* oral squamous cell carcinoma; *HNSCC* head and neck squamous cell carcinoma; *LSCC* laryngeal squamous cell carcinoma; *OR* odds ratio; *CI* confidence interval

### COX-2 rs689466 polymorphism and HNSCC risk

**Fig. 2:**
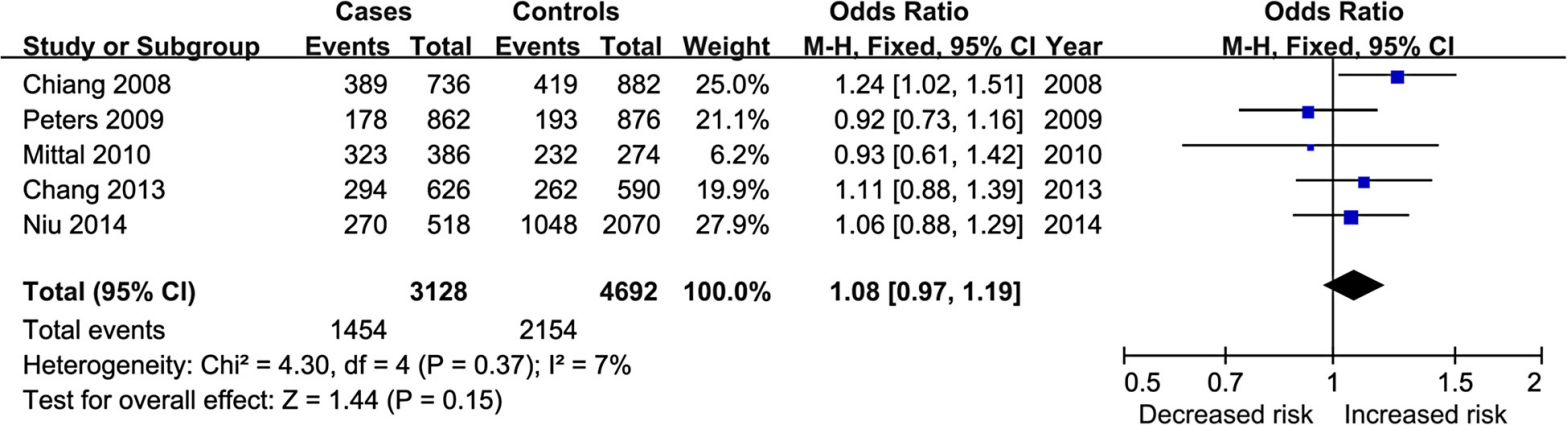
Forest plot for A vs. G model of crude data of rs689466 polymorphism

The pooled results from crude data indicated there was a significant increased risk of association between COX-2 rs689466 polymorphism and HNSCC risk in AA vs. GG, AA vs. GA, and AA vs. GG + GA genetic models while no association in A vs. G (Fig. 2) and AA + GA vs. GG genetic models. Subgroup analyses stratified by ethnicity and cancer site all revealed negative results. The results of adjusted data showed no association between COX-2 rs689466 polymorphism and HNSCC risk in overall population and subgroup analyses. The sensitivity analysis showed the results without substantive change. Table 3 showed the results of all analyses.

**Table 3:** Overall and subgroups meta-analysis of COX-2 rs689466 polymorphism and HNSCC risk

Overall and subgroups	No.	OR (95 % CI)	Heterogeneity (*I* ^2^%/p)
A vs. G (unadjusted and adjusted)			
Overall (unadjusted)	5	1.08 (0.97–1.09)	7 %/0.37
Overall (adjusted)	2	1.07 (0.84–1.36)	0 %/0.88
Asians (unadjusted)	4	1.12 (1.00–1.25)	0 %/0.56
Asians (adjusted)	2	1.07 (0.84–1.36)	0 %/0.88
Caucasian (unadjusted)	1	0.92 (0.73–1.16)	NA
OSCC (unadjusted)	3	1.01 (0.87–1.16)	80 %/0.008
OSCC (adjusted)	1	1.03 (0.60–1.42)	NA
LSCC (unadjusted)	1	0.96 (0.72–1.32)	NA
AA vs. GG (unadjusted)			
Overall	5	1.26 (1.01–1.57)	0 %/0.46
Asians	4	1.25 (0.99–1.57)	14 %/0.32
Caucasian	1	1.39 (0.70–2.73)	NA
OSCC	3	1.07 (0.40–2.86)	86 %/<0.05
LSCC	1	1.30 (0.69–2.45)	NA
AA vs. GA (unadjusted and adjusted)			
Overall (unadjusted)	5	1.21 (1.01–1.45)	28 %/0.23
Overall (adjusted)	4	0.84 (0.69–1.03)	0 %/0.41
Asians (unadjusted)	4	1.17 (0.97–1.42)	30 %/0.23
Asians (adjusted)	3	0.89 (0.68–1.16)	23 %/0.27
Caucasian (unadjusted)	1	1.78 (0.89–3.57)	NA
Caucasian (adjusted)	1	0.79 (0.58–1.07)	NA
OSCC (unadjusted)	3	0.88 (0.53–1.48)	76 %/0.01
OSCC (adjusted)	2	1.23 (0.23–4.70)	67 %/0.08
LSCC (unadjusted)	1	1.17 (0.71–1.93)	NA
LSCC (adjusted)	1	1.16 (0.65–2.09)	NA
AA vs. GG + GA (unadjusted)			
Overall	5	1.20 (1.01–1.43)	12 %/0.34
Asians	4	1.18 (0.99–1.41)	26 %/0.26
Caucasian	1	1.52 (0.78–2.96)	NA
OSCC	3	0.89 (0.50–1.58)	83 %/0.003
LSCC	1	1.21 (0.75–1.94)	NA
AA + GA vs. GG (unadjusted and adjusted)			
Overall (unadjusted)	5	0.98 (0.84–1.15)	28 %/0.23
Overall (adjusted)	2	0.93 (0.72–1.21)	0 %/0.84
Asians (unadjusted)	4	1.07 (0.88–1.29)	13 %/0.33
Asians (adjusted)	2	0.93 (0.72–1.21)	0 %/0.84
Caucasian	1	0.83 (0.63–1.09)	NA
OSCC (unadjusted)	3	1.03 (0.57–1.88)	75 %/0.02
OSCC (adjusted)	1	0.78 (0.53–1.14)	NA
LSCC (unadjusted)	1	1.17 (0.68–2.03)	NA
LSCC (adjusted)	1	1.23 (0.71–2.15)	NA

*OSCC* oral squamous cell carcinoma; *HNSCC* head and neck squamous cell carcinoma; *LSCC* laryngeal squamous cell carcinoma; *OR* odds ratio; *CI* confidence interval; *NA* not available

### COX-2 rs5275 polymorphism and HNSCC risk

**Fig. 3:**
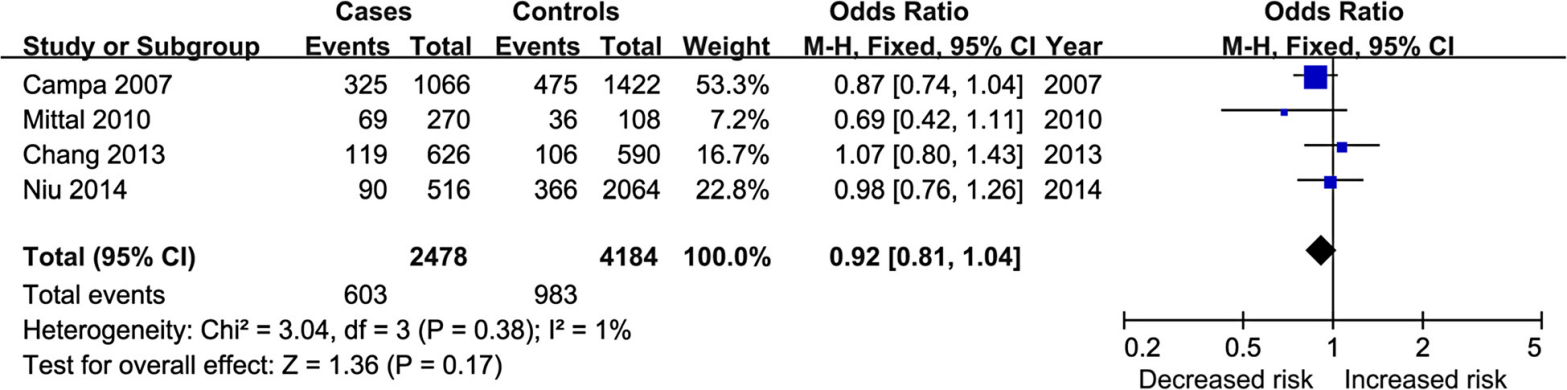
Forest plot for C vs. T model of crude data of rs5275 polymorphism

The pooled results of crude and adjusted data all showed nonsignificant association between COX-2 rs5275 polymorphism and HNSCC risk in overall population, Fig. 3 showed the result of C vs. T model of crude data. The results of subgroup analyses all revealed negative association. The sensitivity analysis showed the results without substantive change. Table 4 showed the results of all analyses.

**Table 4:** Overall and subgroups meta-analysis of COX-2 rs5275 polymorphism and HNSCC risk

Overall and subgroups	No.	OR (95 % CI)	Heterogeneity (*I* ^2^%/p)
C vs. T (unadjusted and adjusted)			
Overall (unadjusted)	4	0.92 (0.81–1.04)	1 %/0.38
Overall (adjusted)	2	1.06 (0.81–1.40)	0 %/0.33
HWE (Yes–unadjusted)	3	0.94 (0.82–1.06)	0 %/0.45
HWE (No–unadjusted)	1	0.69 (0.42–1.11)	NA
Asians (unadjusted)	3	0.97 (0.87–1.16)	17 %/0.30
Asians (adjusted)	2	1.06 (0.81–1.40)	0 %/0.33
Caucasian (unadjusted)	1	0.87 (0.74–1.04)	NA
OSCC (unadjusted)	3	0.90 (0.76–1.06)	0 %/0.52
OSCC (adjusted)	1	0.88 (0.55–1.40)	NA
LSCC (unadjusted)	2	0.88 (0.73–1.06)	47 %/0.17
CC vs. TT (unadjusted and adjusted)			
Overall (unadjusted)	4	0.92 (0.67–1.27)	36 %/0.19
Overall (adjusted)	4	0.92 (0.67–1.27)	49 %/0.12
HWE (Yes–unadjusted)	3	0.89 (0.64–1.24)	47 %/0.15
HWE (No–unadjusted)	1	2.59 (0.31–21.82)	NA
Asians (unadjusted)	3	1.49 (0.87–2.57)	0 %/0.86
Asians (adjusted)	3	1.45 (0.81–2.59)	16 %/0.30
Caucasian (unadjusted)	1	0.71 (0.47–1.07)	NA
Caucasian (adjusted)	1	0.75 (0.51–1.10)	NA
OSCC (unadjusted)	3	0.92 (0.59–1.43)	0 %/0.48
OSCC (adjusted)	3	0.89 (0.56–1.40)	0 %/0.57
LSCC (unadjusted)	2	0.98 (0.35–2.75)	67 %/0.08
LSCC (adjusted)	2	0.88 (0.34–2.26)	60 %/0.12
CC vs. CT (unadjusted and adjusted)			
Overall (unadjusted)	4	1.02 (0.74–1.41)	48 %/0.12
Overall (adjusted)	4	0.99 (0.84–1.16)	0 %/0.60
HWE (Yes–unadjusted)	3	0.96 (0.68–1.33)	42 %/0.18
HWE (No–unadjusted)	1	5.13 (0.61–42.88)	NA
Asians (unadjusted)	3	1.73 (0.99–3.01)	0 %/0.54
Asians (adjusted)	3	0.93 (0.73–1.19)	0 %/0.49
Caucasian (unadjusted)	1	0.77 (0.52–1.16)	NA
Caucasian (adjusted)	1	1.03 (0.82–1.28)	NA
OSCC (unadjusted)	3	0.99 (0.64–1.53)	44 %/0.17
OSCC (adjusted)	3	1.02 (0.80–1.30)	28 %/0.25
LSCC (unadjusted)	2	0.87 (0.54–1.40)	50 %/0.16
LSCC (adjusted)	2	0.92 (0.70–1.21)	0 %/0.62
CC vs. CT + TT (unadjusted)			
Overall	4	0.96 (0.70–1.31)	43 %/0.15
HWE (Yes)	3	0.91 (0.66–1.25)	46 %/0.16
HWE (No)	1	3.65 (0.45–29.89)	NA
Asians	3	1.58 (0.93–2.71)	0 %/0.70
Caucasian	1	0.74 (0.50–1.09)	NA
OSCC	3	0.94 (0.62–1.43)	16 %/0.30
LSCC	2	1.02 (0.40–2.60)	63 %/0.10
CC + CT vs. TT (unadjusted and adjusted)			
Overall (unadjusted)	4	0.90 (0.77–1.04)	0 %/0.41
Overall (adjusted)	3	0.98 (0.84–1.15)	0 %/0.78
HWE (Yes–unadjusted)	3	0.92 (0.79–1.08)	0 %/0.74
HWE (No–unadjusted)	1	0.57 (0.30–1.05)	NA
Asians (unadjusted)	3	0.91 (0.74–1.12)	29 %/0.25
Asians (adjusted)	2	1.00 (0.79–1.27)	0 %/0.49
Caucasian (unadjusted)	1	0.88 (0.70–1.10)	NA
Caucasian (adjusted)	1	0.97 (0.78–1.20)	NA
OSCC (unadjusted)	3	1.09 (0.55–2.16)	91 %/<0.05
OSCC (adjusted)	2	1.01 (0.80–1.27)	2 %/0.31
LSCC (unadjusted)	2	0.87 (0.68–1.10)	7 %/0.30
LSCC (adjusted)	2	0.89 (0.69–1.15)	0 %/0.35

*OSCC* oral squamous cell carcinoma; *HNSCC* head and neck squamous cell carcinoma; *LSCC* laryngeal squamous cell carcinoma; *OR* odds ratio; *CI* confidence interval; *NA* not available; *HWE* Hardy–Weinberg Equilibrium

### COX-2 rs20417 polymorphism and HNSCC risk

**Fig. 4:**
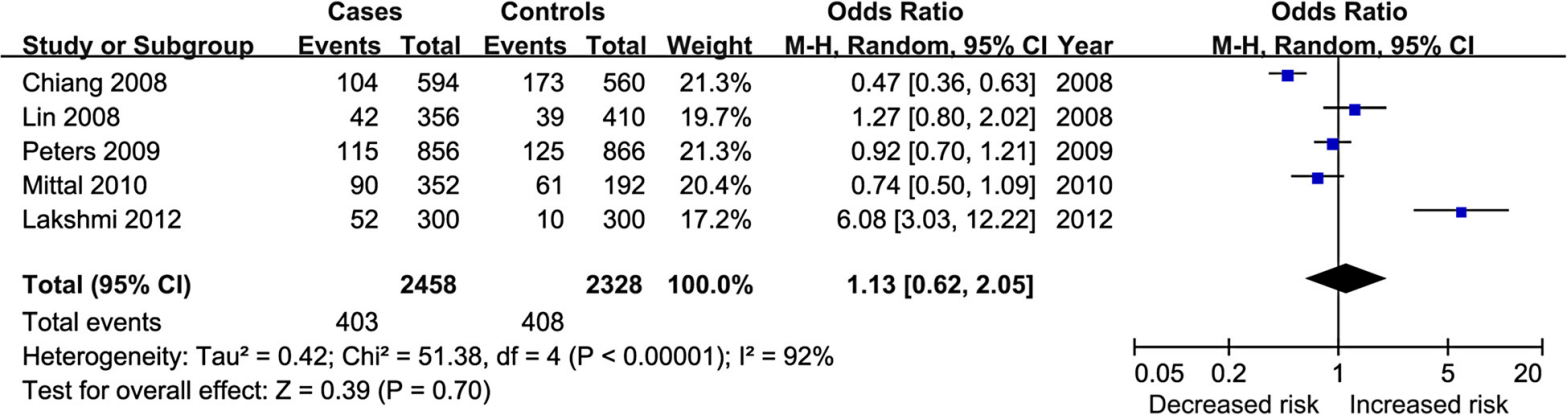
Forest plot for C vs. G model of crude data of rs20417 polymorphism

Table 5 presented the results of COX-2 rs20417 polymorphism and HNSCC risk. All results from unadjusted data and adjusted data presented nonsignificant association, either in overall or subgroups population; Fig. 4 showed the result of C vs. G model of crude data. The sensitivity analysis showed the results without substantive change.

**Table 5:** Overall and subgroups meta-analysis of COX-2 rs20417 polymorphism and HNSCC risk

Overall and subgroups	No.	OR (95 % CI)	Heterogeneity (*I* ^2^%/p)
C vs. G (unadjusted and adjusted)			
Overall (unadjusted)	5	1.13 (0.62–2.05)	92 %/<0.10
OSCC (unadjusted)	4	1.22 (0.52–2.89)	94 %/<0.10
OSCC (adjusted)	1	0.73 (0.50–1.08)	NA
Asians (unadjusted)	4	1.22 (0.52–2.89)	94 %/<0.10
Caucasian (unadjusted)	1	0.92 (0.70–1.21)	NA
HWE (Yes–unadjusted)	4	0.78 (0.52–1.18)	83 %/<0.10
HWE (No–unadjusted)	1	6.08 (3.03–12.22)	NA
CC vs. GG (unadjusted and adjusted)			
Overall (unadjusted)	5	1.17 (0.25–5.46)	80 %/<0.10
Overall (adjusted)	2	0.53 (0.25–1.11)	0 %/0.71
OSCC (unadjusted)	4	1.79 (0.10–31.00)	89 %/<0.10
OSCC (adjusted)	1	0.44 (0.13–1.46)	NA
Asians (unadjusted)	4	1.79 (0.10–31.00)	89 %/<0.10
Caucasian (unadjusted)	1	0.62 (0.25–1.50)	NA
HWE (Yes–unadjusted)	4	0.55 (0.27–1.13)	0 %/0.67
HWE (No–unadjusted)	1	7.75 (1.70–35.33)	NA
GC vs. GG (unadjusted and adjusted)			
Overall (unadjusted)	5	0.69 (0.36–1.35)	0 %/0.75
Overall (adjusted)	2	0.90 (0.68–1.19)	9 %/0.29
OSCC (unadjusted)	4	0.80 (0.30–2.09)	0 %/0.50
OSCC (adjusted)	1	0.71 (0.42–1.18)	NA
Asians (unadjusted)	4	0.80 (0.30–2.09)	0 %/0.50
Caucasian (unadjusted)	1	0.62 (0.24–1.55)	NA
HWE (Yes–unadjusted)	4	0.62 (0.30–1.29)	0 %/0.98
HWE (No–unadjusted)	1	1.29 (0.23–7.31)	NA
CC vs. CG + GG (unadjusted)			
Overall	5	1.15 (0.29–4.54)	76 %/0.02
OSCC	4	1.76 (0.15–21.30)	85 %/<0.10
Asians	4	1.76 (0.15–21.30)	85 %/<0.10
Caucasian	1	0.62 (0.25–1.50)	NA
HWE (Yes)	4	0.58 (0.29–1.18)	0 %/0.84
HWE (No)	1	6.43 (1.41–29.27)	NA
CC + CG vs. GG (unadjusted and adjusted)			
Overall (unadjusted)	5	1.07 (0.51–2.24)	93 %/<0.10
OSCC (unadjusted)	4	1.13 (0.39–3.26)	95 %/<0.10
OSCC (adjusted)	1	0.22 (0.12–0.39)	NA
Asians (unadjusted)	4	1.13 (0.39–3.26)	95 %/<0.10
Caucasian (unadjusted)	1	0.90 (0.70–1.30)	NA
HWE (Yes–unadjusted)	4	0.72 (0.39–1.33)	90 %/<0.10
HWE (No–unadjusted)	1	6.45 (2.90–14.35)	NA

*OSCC* oral squamous cell carcinoma; *HNSCC* head and neck squamous cell carcinoma; *LSCC* laryngeal squamous cell carcinoma; *OR* odds ratio; *CI* confidence interval; *NA* not available; *HWE* Hardy–Weinberg Equilibrium

### Publication bias

Due to the limited number of included studies, we did not conduct publication bias analysis.

## Discussion

The rs20417, rs689466, and rs5275 polymorphisms are the three commonly investigated polymorphisms in the COX-2 gene [14, 15]. In 2007, Campa D et al. conducted a case–control study including 533 cases and 1066 controls which indicated no significant association between COX-2 rs5275 polymorphism and HNSCC risk [27]. Then Chiang SL et al., in 2008, showed that COX-2 rs20417 polymorphism was not associated with OSCC risk but COX-2 rs689466 was associated with increased risk of OSCC [28]. However, another study obtained this increased risk between COX-2 rs20417 polymorphism and OSCC [29]. Similarly, published studies on these three polymorphisms revealed inconsistent results. This meta-analysis based on the crude data indicated there might be an association with increased risk of HNSCC in COX-2 rs689466 polymorphism, but identified negative association between COX-2 rs5275 and COX-2 rs20417 polymorphisms and HNSCC risk. However, the combined results of adjusted data all yielded nonsignificant associations between these three polymorphisms and HNSCC risk. The subgroup analyses according to ethnicity and sites of HNSCC confirm this negative association.

This meta-analysis is the first study to investigate these three polymorphisms and risk of HNSCC. Unlike the usual method, based on unadjusted data [7, 8, 13, 14, 35–38], we also extracted the adjusted data and pooled them for investigating the interactions between genetic polymorphisms and environmental risk factors. Interestingly, the unadjusted data showed COX-2 rs689466 polymorphism might play a role in increased risk while the adjusted data showed a negative association. As we know, smoking and alcohol are the well known risk factors for HNSCC [2, 4]. One study by Mittal M et al. [31] adjusted age and gender only, while the other included studies all adjusted smoking and alcohol. While, there is a relevant meta-analysis by Zhao F et al. published in 2014 [39]. This meta-analysis focused on the association between COX-2 rs20417 polymorphism and digestive system cancer, including three studies of HNSCC [28, 29, 31] and revealed negative association based on the performance of 2 genetic models (GG + GC vs. GG: OR = 0.66, 95 % CI = 0.29, 1.50; C vs. G: OR = 0.95, 95 % CI = 0.56, 1.63). Whereas, our meta-analysis performed all recommended 5 genetic models, included more studies, and considered adjusted data. Furthermore, our meta-analysis investigated 3 polymorphisms at the same time and only focussed on HNSCC. Different cancers have their own histological characteristics and of course their own predisposing genes. The identical polymorphism in the same gene, different polymorphisms in the same gene, and identical polymorphism in different genes might reveal different associations in different cancers. Hence, our meta-analysis was more useful for reference. Also considering this point, we extracted the data for OSCC and LSCC if applicable. The results of all genetic models all showed negative association of OSSS, LSCC, and overall population. In addition we considered genetic background. We stratified the population by ethnicity to explore whether different ethnicities have different susceptibility. The results showed all these 3 polymorphisms in COX-2 gene regardless of genetic background of HNSCC.

As we know, COX-2 participated in cell proliferation and tumour microenvironment and associated with many types of cancer. However, our results showed there was non-association of COX-2 and HNSCC. The possible mechanism of the negative result due to the relative small sample size, which is not enough to detect the small genetic effect. Moreover, COX-2 gene polymorphisms were really not associated with HNSCC risk. Third, the compromise effect might be existed in the 3 polymorphisms of COX-2 or other environmental risk factors, such as green tea. Besides, the haplotype analysis was not performed because of limited information of included studies. However, to explore the true effects and possible mechanism between them remain necessary.

Heterogeneity is 1 of the important issues in genetic association meta-analysis. This limitation also existed in the present meta-analysis, some genetic models showed clear homogeneity while some showed heterogeneity, either in overall population or subgroup analyses (Tables 3, 4 and 5). The heterogeneity might be originated from different genotyping methods, environmental differences, or different lifestyles. However, we could not explore these factors due to the lack of individual data. Also, the number of eligible studies and sample sizes of for each polymorphism was insufficient. Statistical power is influenced by small sample sizes so owing to this limitation, we could not perform publication bias of any polymorphism. We did not confirm whether relevant publications published in languages other than English or Chinese existed, due to lack of right to search and ability to read, as such we may have missed some eligible studies. This limitation was also revealed in the included population. Our meta-analysis only included Asians and Caucasians, hence, our results had no value for other ethnicities. Finally, lacking a relevant recommended tool, we could not assess the methodological quality of included studies and did not performed subgroup analysis based on high vs. low quality. As such, we did not conduct the meta-regression of methodological quality.

## Conclusion

In summary, our meta-analysis based on crude and adjusted data showed that none of COX-2 rs689466, rs5275, and rs20417 polymorphisms was associated with risk of HNSCC. Due to limitations of our meta-analysis, such as insufficient sample sizes, our results should be treated with caution. We recommend further high quality studies, with large sample sizes and stratified by smoking status and alcohol consumption, be conducted to provide high level evidence for clinical implication.

## Abbreviations

CI, confidence interval; COX-2, cyclooxygenase-2; HNSCC, Head and neck squamous cell carcinoma; HPV, Human papillomavirus; HWE, Hardy–Weinberg Equilibrium; LSXX, laryngeal squamous cell carcinoma; OR, Odds ratio; OSCC, Oral squamous cell carcinoma; PRISMA, Preferred Reporting Items for Systematic Reviews and Meta-Analyses; RevMan, Review Manager
